# Information Geometry of Spatially Periodic Stochastic Systems

**DOI:** 10.3390/e21070681

**Published:** 2019-07-12

**Authors:** Rainer Hollerbach, Eun-jin Kim

**Affiliations:** 1 Department of Applied Mathematics, University of Leeds, Leeds LS2 9JT, UK; 2 School of Mathematics and Statistics, University of Sheffield, Sheffield S3 7RH, UK

**Keywords:** stochastic processes, Fokker–Planck equation, information length

## Abstract

We explore the effect of different spatially periodic, deterministic forces on the information geometry of stochastic processes. The three forces considered are $f_{0}=\sin\left(\pi x\right)/\pi$ and $f_{\pm }\;=\;\sin\left(\pi x\right)/\pi \pm \sin\left(2\pi x\right)/2\pi$, with $f_{-}$ chosen to be particularly flat (locally cubic) at the equilibrium point x=0, and $f_{+}$ particularly flat at the unstable fixed point x=1. We numerically solve the Fokker–Planck equation with an initial condition consisting of a periodically repeated Gaussian peak centred at x=μ, with μ in the range [0,1]. The strength *D* of the stochastic noise is in the range $10^{-4}$–$10^{-6}$. We study the details of how these initial conditions evolve toward the final equilibrium solutions and elucidate the important consequences of the interplay between an initial PDF and a force. For initial positions close to the equilibrium point x=0, the peaks largely maintain their shape while moving. In contrast, for initial positions sufficiently close to the unstable point x=1, there is a tendency for the peak to slump in place and broaden considerably before reconstituting itself at the equilibrium point. A consequence of this is that the information length $L_{\infty }$, the total number of statistically distinguishable states that the system evolves through, is smaller for initial positions closer to the unstable point than for more intermediate values. We find that $L_{\infty }$ as a function of initial position μ is qualitatively similar to the force, including the differences between $f_{0}=\sin\left(\pi x\right)/\pi$ and $f_{\pm }=\sin\left(\pi x\right)/\pi \pm \sin\left(2\pi x\right)/2\pi$, illustrating the value of information length as a useful diagnostic of the underlying force in the system.

## 1. Introduction

It is of interest to apply the idea of a metric to problems involving stochastic processes, e.g., [1,2,3,4,5,6]. Given a metric, the differences between different Probability Density Functions (PDFs) can be quantified, with different metrics focusing on a range of aspects, and hence most suitable for various applications. Fisher information [7] yields a metric where distance is measured in units of the PDF’s width. The distance in the Fisher metric is thus dimensionless, and represents the number of statistically different states [8].

By extending the statistical distance in [8] to time-dependent situations, we recently introduced a way of quantifying information changes associated with time-varying PDFs [9,10,11,12,13,14,15,16]. We first compare two PDFs separated by an infinitesimal increment in time, and consider the corresponding infinitesimal distance. Integrating in time gives the total number of statistically distinguishable states that the system passes through, called the *information length* L, e.g., [6,7,8,14]. Another interpretation of L that can be useful is as a measure of the total elapsed time in units of an ‘information-change’ dynamical timescale.

We start by defining the dynamical time τ(t) as

(1) $$E\equiv \frac{1}{{\left[\tau \left(t\right)\right]}^{2}}=\int \frac{1}{p(x,t)}{\left[\frac{\partial p(x,t)}{\partial t}\right]}^{2}\;dx.$$

That is, τ(t) is the characteristic timescale over which the information changes, and quantifies the PDF’s correlation time. Alternatively, 1/τ quantifies the (average) rate of change of information in time. A PDF that evolves such that E is constant in time is referred to as a geodesic, along which the information propagates at a uniform rate [6]. The information length L(t) is then defined by

(2) $$L\left(t\right)=\int_{0}^{t}\frac{dt_{1}}{\tau (t_{1})}=\int_{0}^{t}\sqrt{\int dx\frac{1}{p(x,t_{1})}{\left[\frac{\partial p(x,t_{1})}{\partial t_{1}}\right]}^{2}}\;dt_{1}.$$

which can be interpreted as measuring time in units of τ. It is important to note that L has no dimension (unlike entropy) and represents the total number of statistically different states that a system passes through in time between 0 and *t*. If we know the parameters that determine the PDF p(x,t), E and L in Equations (1) and (2) can be written in terms of the Fisher metric tensor defined in the statistical space spanned by those parameters. However, it is not always possible to have access to the parameters that govern PDFs, for instance, in the case of PDFs calculated from data. The merit of Equations (1) and (2) is thus that E and L can be directly calculated from PDFs even without knowing the parameters governing the PDFs, nor the Fisher metric. For instance, L was calculated from PDFs of music data in [10]. In the work here, we first compute time-dependent PDFs by solving the Fokker–Planck equation numerically, and then calculate E and L from these PDFs as additional diagnostics.

Unlike quantities such as entropy, relative entropy, Kullback–Leibler divergence, or Jensen divergence, information length is a Lagrangian measure, that is, it includes the full details of the PDF’s evolution, and not just the initial and final states. $L_{\infty }$, the total information length over the entire evolution, is then particularly useful to quantify the proximity of any initial PDF to a final attractor of a dynamical system. In previous work [12,15] we explored these aspects of L for restoring forces that were power-laws in the distance to the attractor. For instance, for the Ornstein–Uhlenbeck process, which is a linear relaxation process, we showed that L consists of two parts: the first is due to the movement of the mean position measured in units of the width of the PDF, and the second is due to the entropy change. Thus, the total entropy change that is often discussed in previous works (e.g., [17]) contributes only partially to L. Importantly, for the Ornstein–Uhlenbeck process, $L_{\infty }$ increases linearly from the stable equilibrium point (with its minimum value at the stable equilibrium point) with the mean position of the initial PDFs regardless of the strength of the stochastic noise and the width of the initial PDFs. The linear relation indicates that a linear process preserves a linearity of the underlying process. Heseltine & Kim [18] shows that this linear relation is lost for other metrics (e.g., Kullback–Leibler divergence, Jensen divergence). Note that L is related to the integral of the square root of the infinitesimal relative entropy (see Appendix A). In comparison, for a chaotic attractor, $L_{\infty }$ varies sensitively with the mean position of a narrow initial PDF, taking its minimum value at the most unstable point [9]. This sensitive dependence of $L_{\infty }$ on the initial PDF is similar to a Lyapunov exponent.

These results highlight $L_{\infty }$ as an alternative diagnostic to understand attractor structures of dynamical systems. It is this attractor structure that we are interested in in this paper. We thus focus on the relaxation problem as in [9,12,15,18] by considering periodic deterministic forces and elucidate the importance of the initial condition and its interplay with the deterministic forces in the relaxation and thus attractor structure.

## 2. Model

We consider the following nonlinear Langevin equation:

(3) $$\frac{dx}{dt}=-f\left(x\right)+\xi .$$

Here *x* is a random variable; f(x) is a deterministic force; ξ is a stochastic forcing, which for simplicity can be taken as a short-correlated Gaussian random forcing as follows:

(4) $$\langle \xi \left(t\right)\xi \left(t^{\prime }\right)\rangle =2D\delta \left(t-t^{\prime }\right),$$

where the angular brackets represent the average over ξ, 〈ξ〉=0, and *D* is the strength of the forcing.

In [15] we considered the choice $f\left(x\right)=x^{n}$ and investigated how varying the degree of nonlinearity n=3,5,7 affects the system. In this work we take f(x) to be periodic in *x*, and explore some of the new effects this can create. The three choices of f(x) we consider are

(5) $$f_{0}=\sin\left(\pi x\right)/\pi ,\;f_{\pm }=\sin\left(\pi x\right)/\pi \pm \sin\left(2\pi x\right)/2\pi .$$

**Figure 1:**
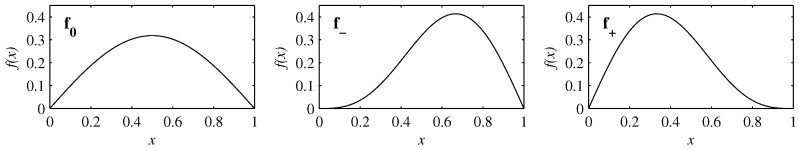
The three profiles $f_{0}$, $f_{-}$ and $f_{+}$. Note how $f_{-}$ is chosen to be flat at the attractor x=0, and $f_{+}$ at the unstable fixed point x=1. All three choices are anti-symmetric in *x*, and periodic with period 2.

Figure 1 shows these profiles, which are all anti-symmetric in *x*, and periodic on the interval x∈[-1,1]. All three choices have x=0 as an attractor, and x=1 as an unstable fixed point. The particular combinations of harmonics for $f_{\pm }$ were chosen so that they are locally cubic rather than linear at either x=0 (for $f_{-}$) or x=1 (for $f_{+}$). In applications such a Brownian motors many specific choices of f(x) are considered to model particular physics. However, as noted in the introduction, we are here more interested in attractor structures in the relaxation problem, in particular, how initial conditions and stochastic noise interact with deterministic forces and the role of the asymmetry of the deterministic force and the stable and unstable fixed points on the local dynamics.

Comparing these three periodic functions with the previous choices, two significant differences stand out. First, for $f\left(x\right)=x^{n}$ with n=3,5,7, all initial conditions are pushed directly toward the origin, and there are no unstable fixed points. It is therefore of particular interest to see how the choices here behave for initial conditions near x=1. Second, $f\left(x\right)=x^{3,5,7}$ all curve upward (that is, have $f^{\prime \prime }>0$ for all x>0), whereas the choices here have different combinations of curvatures, which will turn out to have clearly identifiable effects.

The Fokker–Planck equation [19,20] corresponding to Equation (3) is

(6) $$\partial_{t}p\left(x,t\right)=\partial_{x}\left(f\left(x\right)p\right)+D\partial_{xx}p.$$

In [15] we solved the corresponding equation by finite-differencing in *x*. For the periodic systems considered here, it is more convenient to start with the Fourier expansion

(7) $$p\left(x,t\right)=a_{0}\left(t\right)+\sum_{k=1}^{K}\left(a_{k}\left(t\right)\cos\left(k\pi x\right)+b_{k}\left(t\right)\sin\left(k\pi x\right)\right).$$

The coefficients $a_{k}$ and $b_{k}$ are then time-stepped using second-order Runge-Kutta. The term $\partial_{x}\left(f\left(x\right)p\right)$ is separated out into the relevant Fourier components using a fast Fourier transform. (For the very simple choices of f(x) considered here, consisting of at most two Fourier modes, it would be straightforward to do this separation analytically, and thereby do the entire calculation purely in Fourier space, but the code was developed with more general choices for f(x) in mind, where this approach becomes increasingly cumbersome as the number of harmonics in f(x) increases. For such more general choices of f(x) the FFT approach is most convenient).

Resolutions in the range $K=2^{11}-2^{14}$ are used, and carefully checked to ensure fully resolved solutions. Time-steps were in the range $10^{-4}$–$10^{-5}$, and were again varied to ensure proper accuracy. Another useful test of the numerical implementation is to monitor the coefficient $a_{0}$: this is time-stepped along with the others, but must in fact remain constant if the total probability $\int p\;dx=2a_{0}$ is to remain constant. It was found that if the initial condition is correctly set to have $a_{0}=0.5$, then this was maintained throughout the entire subsequent evolution.

The initial conditions are of the form

(8) $$p\left(x,0\right)=\frac{1}{\sqrt{2\pi \;D_{0}}}\exp\left[-\frac{{\left(x-\mu \right)}^{2}}{2D_{0}}\right],$$

that is, Gaussians centred at x=μ and having half-width scaling as $\sqrt{D_{0}}$. We are interested in the range μ∈[0,1]; by symmetry the range μ∈[-1,0] would behave the same, simply approaching x=0 from the other direction.

This initial condition is also periodic, on the same x∈[-1,1] interval as the entire problem. For the purposes of actually implementing Equation (8), it was most convenient to consider the range as being x∈[-0.5,1.5]. In particular, for μ∈[0,1] and the values of $D_{0}$ considered here, Equation (8) yields results at x=-0.5 and x=1.5 that are different, but both are so vanishingly small that the discrepancy does not need to be smoothed out in defining the initial condition. If instead Equation (8) were implemented on either x∈[0,2] or x∈[-1,1], then μ near either 0 or 1 would be more awkward to handle correctly.

In [15] we also used a Gaussian initial condition, with $D_{0}=10^{-6}$, and then explored the regime $D=10^{-6}$ to $10^{-9}$. Here we are again interested in the regime $D_{0}\geq D$, which allows at least the initial parts of the evolution to be nondiffusive. Having the initial peak be so narrow that $D_{0}<D$ can also be interesting in other contexts (e.g., [21]), but diffusive effects are then necessarily important from the outset, which would obscure some of the dynamics of interest here. We therefore focus on the range $D_{0}=10^{-2}$ to $10^{-4}$, and $D=10^{-4}$ to $10^{-6}$.

## 3. Results

**Figure 2:**
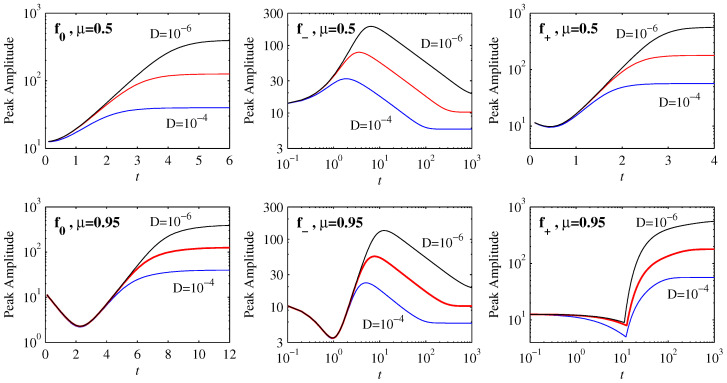
Peak amplitudes as functions of time, for the three choices $f_{*}$ as labelled. The initial positions are at μ=0.5 in the top row, and μ=0.95 in the bottom row, with initial widths $D_{0}=10^{-3}$ in all cases. $D=10^{-4}$ to $10^{-6}$ as labelled, also colour-coded as blue, red, black. Note also that some panels have *t* on a linear scale, indicating a very rapid adjustment process, whereas others have *t* on a logarithmic scale, corresponding to much slower dynamics.

Figure 2 shows how the peak amplitudes evolve in time for the three choices $f_{0}$, $f_{-}$ and $f_{+}$. Starting with the initial position μ=0.5 in the top row, we see that the solutions for $f_{0}$ and $f_{+}$ equilibrate to their final values on very rapid timescales, involving relatively little variation with *D*. In contrast, the timescales for $f_{-}$ are much longer, and vary substantially with *D*. Comparing the $f_{-}$ results here with Figure 1 in [15], we see that $f_{-}$ is exactly analogous to the previous $f\left(x\right)=x^{3}$. This is because for x≤0.5 the shape of $f_{-}$ is very close to a cubic. Similarly, for x≤0.5 the shape of $f_{0}$ is still reasonably close to linear, and the evolution is therefore essentially like the linear Ornstein–Uhlenbeck process f(x)=x, for which an exact analytic solution exists [21].

It is only $f_{+}$ whose shape is already substantially different from either linear or cubic even on the interval x≤0.5, being close to linear for x≤0.2 but strongly curved for 0.2<x≤0.5. Correspondingly $f_{+}$ also shows a new effect, namely an initial reduction in the peak amplitudes. This effect becomes even more pronounced for $f_{0}$ and $f_{-}$ and the initial position μ=0.95, in the bottom row of Figure 2. This reduction in the peak amplitudes is *not* caused by diffusive spreading but is a consequence of the non-diffusive (ξ=D=0) evolution resulting from the interplay between an initial PDF and the deterministic force. We note in particular how $D=10^{-4}$ to $10^{-6}$ yield identical reductions in amplitudes here. It is worth comparing this with the non-diffusive evolution in [15] where the opposite behavior—an initial increase in peak amplitudes (the same effect as seen here for $f_{-}$, μ=0.5)—was observed. The interplay between the initial PDF and the deterministic force is elaborated below.

If f(x) is such that it increases more rapidly than linearly, i.e., curves upward, then those parts of any initial condition furthest from the origin are pushed toward it fastest, whereas those parts closest move more slowly. The result is that an initial Gaussian peak bunches up on itself, causing the amplitude to increase. In contrast, if f(x) curves downward the opposite effect occurs, and an initial Gaussian peak is spread out, even before diffusion starts to play a role. Eventually of course the peak moves sufficiently close to the origin that the behaviour is as before, explaining why the behaviour at later times is similar to the previous μ=0.5 results.

Finally, the behaviour for $f_{+}$ with μ=0.95 is yet again different, namely an initial reduction in amplitude up to t≈10, followed by an abrupt increase. This is caused by a fundamentally new peak forming at the origin, rather than the initial peak moving toward it. Note also that time here is on a logarithmic scale, corresponding to a very slow equilibration process, unlike the previous case $f_{+}$ with μ=0.5.

**Figure 3:**
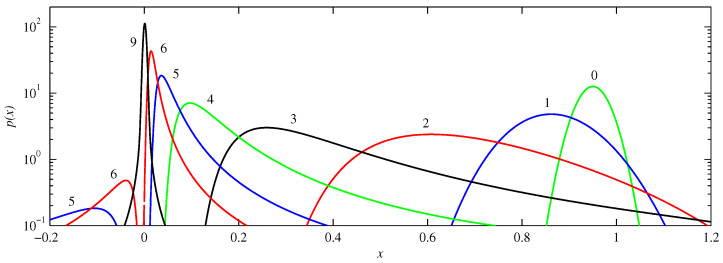
The solutions p(x,t) for $f_{0}$, $D=10^{-5}$, and initial condition μ=0.95 and $D_{0}=10^{-3}$. The numbers beside individual curves indicate the times, from t=0 to 9. The different colours are for clarity only, but do not indicate a specific colour ↔ time relationship.

**Figure 4:**
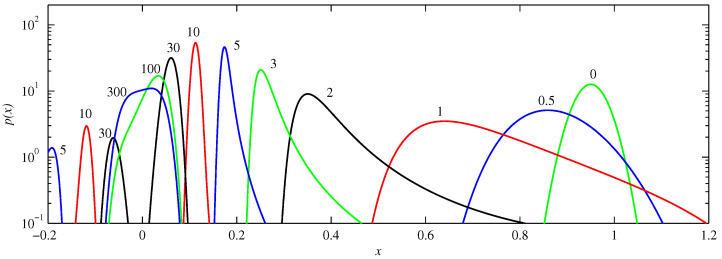
The solutions p(x,t) for $f_{-}$, $D=10^{-5}$, and initial condition μ=0.95 and $D_{0}=10^{-3}$. The numbers beside individual curves indicate the times, from t=0 to 300. Different colours are again only to help distinguish between the different lines.

**Figure 5:**
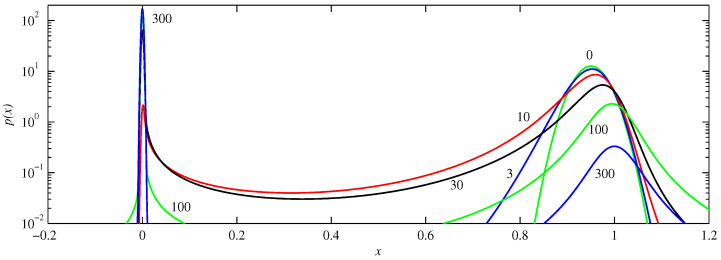
The solutions p(x,t) for $f_{+}$, $D=10^{-5}$, and initial condition μ=0.95 and $D_{0}=10^{-3}$. The numbers beside individual curves indicate the times, from t=0 to 300.

Figure 3, Figure 4 and Figure 5 illustrate these various behaviours in more detail, showing the actual PDFs at different times for $f_{0}$, $f_{-}$ and $f_{+}$, respectively. Starting with $f_{0}$, we see how the peak initially located at μ=0.95 becomes broader as it moves toward the origin, an effect again not caused by diffusion, but rather by the curvature of $f_{0}$ at these values of *x*. Note for example how the solutions at t=3 or 4 have much steeper leading edges (nearer to the origin) than trailing edges, caused by the trailing edges moving so much slower. Another feature to note is how parts of the solution reach the origin coming from the ‘other’ direction. That is, if the initial condition is a peak centred at μ=0.95, and having half-width 0.07 (corresponding to $D_{0}=10^{-3}$), then a small but non-negligible portion of the initial condition is in the range x≥1, as seen also in Figure 3. For this part of the initial condition the nearest attractor is x=2 rather than x=0. Viewed on the interval x∈[-0.2,1.2], this part therefore approaches from negative *x* values, as seen at times t=5 and 6. (The interval x∈[1.2,1.8] or equivalently x∈[-0.8,-0.2] is not shown in these figures because the amplitudes are rather small there, due to the PDFs being very spread out as they traverse this range). Finally, between t=6 and 9 we see how the two peaks coming from negative and positive *x* values combine to form the single final equilibrium consisting of a Gaussian centred at the origin.

Figure 4 shows the corresponding solutions for $f_{-}$. For small durations the behaviour is very similar to that seen in Figure 3, except that it happens roughly twice faster (e.g., compare t=0.5 in Figure 4 with t=1 in Figure 3). This is readily understandable by noting that the slope of $f_{-}$ near x=0.95 is roughly twice that of $f_{0}$, yielding faster evolution. The later evolution is much slower though, with the merging of the two peaks only occurring between t=30 and 100, and even t=300 still displaying some asymmetry, and hence not yet the final quartic profile. This is the same very slow final adjustment process previously analysed in detail in [15], and is caused by $f_{-}$ being cubic rather than linear near the origin.

Figure 5 shows the solutions for $f_{+}$. We see the behaviour alluded to above, of an abrupt transition from one peak to another. Because $f_{+}$ is so flat near x=1, there is hardly any tendency to push the initial peak away. Instead, it simply broadens out, slumping as it spreads. A new peak then forms at the origin, overtaking the original one in amplitude around t≈10, as previously noted in Figure 2. Note though that long after this time a significant portion of the original peak still remains near x=1, and this portion only fades away on very long timescales; x=1 is an unstable fixed point, but $f_{+}$ is so small everywhere near x=1 that there is very little tendency to push the solutions away from there.

**Figure 6:**
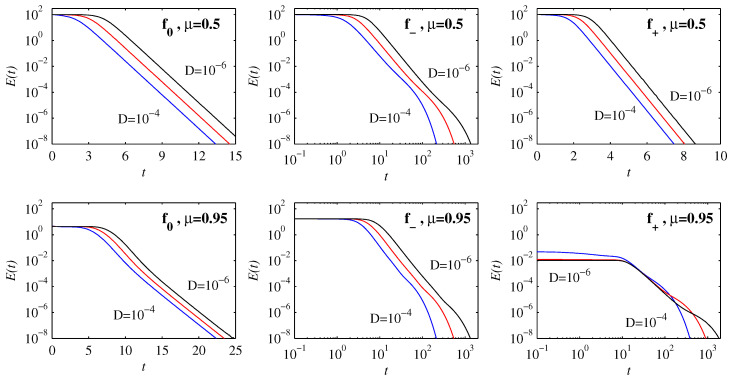
E as a function of time, for the six configurations as in Figure 2. Note again how the scale for *t* is sometimes linear and sometimes logarithmic.

As noted in the introduction, we are particularly interested in the effects that these various different types of behaviour have on the information length quantities E(t) and $L_{\infty }$. Figure 6 shows E(t) for the same solutions as before in Figure 2. We see that E is initially uniform, and independent of *D* (provided *D* is sufficiently small in comparison with $D_{0}$), corresponding to the ‘geodesic’ behaviour first identified by [6]. For some configurations, E then immediately transitions to an exponential decay, whereas for others it first has a power-law decay before ultimately decaying exponentially. Correspondingly, the timescales to achieve $E\leq 10^{-8}$ also vary dramatically, as seen by the various linear and logarithmic scales for *t*. Different scaling regimes signify fundamentally different dynamics.

**Figure 7:**
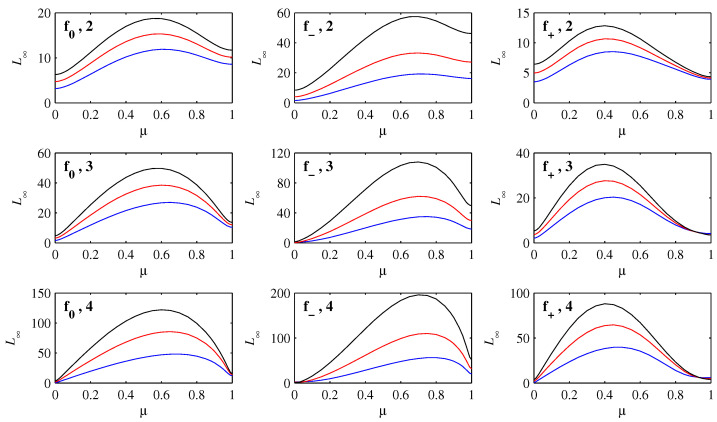
The total information length $L_{\infty }$ as a function of initial position μ. The labels $f_{*},n$ indicate the particular profile $f_{*}$, and the initial width $D_{0}=10^{-n}$. Within each panel the three curves are $D=10^{-4}$ (lowest, blue), $D=10^{-5}$ (middle, red), and $D=10^{-6}$ (top, black).

Figure 7 shows how $L_{\infty }$ varies with μ, for $D_{0}=10^{-2}$ to $10^{-4}$, and $D=10^{-4}$ to $10^{-6}$ within each panel. It is interesting to note how the shapes generally mimic the corresponding functions $f_{0}$, $f_{-}$ and $f_{+}$. The largest values always occur for intermediate values of μ, even though larger values correspond to initial conditions that have farther to travel to reach the origin. Such initial conditions also spread out much more though, as seen above, and according to the interpretation of information length, this should indeed reduce L. Very close to x=1 the $L_{\infty }$ values are particularly small, because having peaks collapse in place and reform at the new location is an informationally very efficient way to move, as seen also in other contexts [13,22,23].

**Figure 8:**
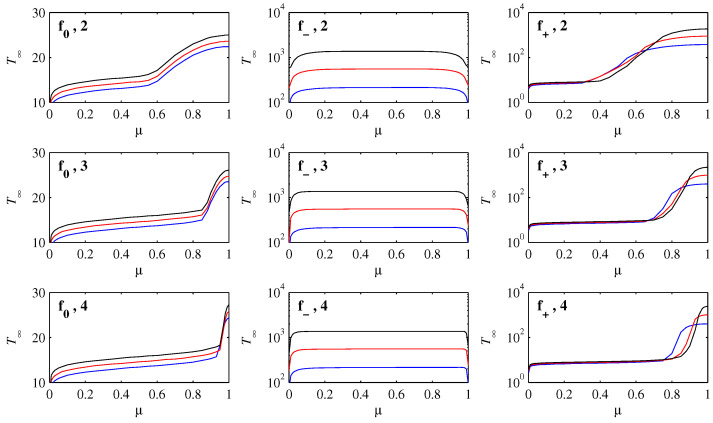
As in Figure 7, but now showing the time $T_{\infty }$ that it takes to achieve $E=10^{-8}$, that is, a measure of the time it takes to reach $L_{\infty }$. Note how $T_{\infty }$ has a linear scale for $f_{0}$, but logarithmic scales for $f_{-}$ and $f_{+}$. The colour-coding is again $D=10^{-4}$ blue, $D=10^{-5}$ red, $D=10^{-6}$ black.

Finally, Figure 8 shows the time, call it $T_{\infty }$, needed for E to drop to $10^{-8}$. The precise cutoff $E=10^{-8}$ is of course somewhat arbitrary, but as seen in Figure 6 is sufficiently small to be in the exponential decay regime in all cases. This is therefore a convenient measure of the time taken to reach $L_{\infty }$, and any even smaller cutoff would only add small increments to $T_{\infty }$ (and essentially nothing to $L_{\infty }$).

Starting with $f_{0}$, we note first that $T_{\infty }$ is on a linear scale, meaning that each reduction of *D* by a factor of 10 only adds a constant amount to $T_{\infty }$. This is the same effect already seen in Figure 2, where smaller *D* requires slightly longer to settle in to the final states. Equivalently, smaller *D* in Figure 6 remains in the flat, geodesic regime for slightly longer times. The other feature to note for $f_{0}$ is the behaviour near μ=1, where $T_{\infty }$ increases strongly, and increasingly abruptly for smaller $D_{0}$. This can be understood by noting that if $\sqrt{D_{0}}\ll 1-\mu$, the initial condition Equation (8) is essentially zero at x=1, whereas if μ is within $\sqrt{D_{0}}$ of 1, Equation (8) does have a non-negligible component at x=1. Therefore, if $\sqrt{D_{0}}\ll 1-\mu$ the initial peak will simply move monotonically toward the origin, which occurs on a rapid timescale, whereas if $1-\mu \leq \sqrt{D_{0}}$ the evolution will include a significant component of the slumping-in-place behaviour, which we saw only happens on slower timescales.

For $f_{-}$, the scale for $T_{\infty }$ is logarithmic, so that each reduction of *D* by a factor of 10 increases $T_{\infty }$ by a factor of $\sqrt{10}$. For intermediate values of μ, $T_{\infty }$ is also essentially independent of μ. The equilibration time is completely dominated by the final settling-in time, just as in the cubic case in [15], and the initial motion of the peak toward the origin is negligible in comparison. For very small values of μ the behaviour is different, with much smaller values of $T_{\infty }$. If $\mu \leq \sqrt{D_{0}}$, the peak is essentially at the origin already, making the adjustment quicker. Finally, there is a similar end-effect for μ sufficiently close to 1; if $1-\mu \leq \sqrt{D_{0}}$, the initial peak is essentially at the unstable fixed point, and the evolution is the slumping-in-place behaviour, which has a faster final adjustment than if the peak moves toward the origin and then adjusts its shape there (but still scaling as $D^{-1/2}$).

Finally, $f_{+}$ is qualitatively similar to $f_{0}$, in the sense that $T_{\infty }$ is a monotonically increasing function of μ. Indeed, for intermediate values of μ the behaviour is virtually identical to $f_{0}$, with $T_{\infty }$ increasing by a constant amount every time *D* is decreased by a constant factor. (This is simply not visible because $T_{\infty }$ is on a logarithmic rather than linear scale here). Because $f_{0}$ and $f_{+}$ are both linear near the origin, the extremely slow final adjustment that happens for $f_{-}$ does not apply to either of them, leaving only this much weaker dependence on *D*. The behaviour near μ=1, with the very strong increase in $T_{\infty }$, and again more abruptly for smaller $D_{0}$, is again because this is the regime where the slumping-in-place behaviour occurs. Also, because $f_{+}$ is so much flatter near x=1 than either of $f_{0}$ of $f_{-}$, this slumping-in-place behaviour is much slower for $f_{+}$ than for the other choices (recall again how long the peak at x=1 lasts in Figure 5). This explains why $T_{\infty }$ is on a logarithmic scale for $f_{+}$ but on a linear scale for $f_{0}$, even though for intermediate values of μ they exhibit the same (weak) scaling with *D*.

## 4. Conclusions

The results presented here extend our previous work [12,15] to the deterministic forces that are periodic in space. This naturally allows for forces f(x) that curve in opposite directions in different regions, as well as unstable fixed points. The deterministic force can also be adjusted to be particularly flat at either the stable equilibrium or the unstable fixed points, which both turn out to have important consequences, with either choice yielding particularly long timescales, scaling as $D^{-1/2}$. The interesting consequences of the interplay between an initial PDF and a deterministic force on the PDF evolution and scalings of different quantities were discussed in detail by considering three types of periodic deterministic forces and comparing the results.

In particular, we computed how the rate of information change and the resulting total information length $L_{\infty }$ depend on the position of an initial Gaussian peak. We found that for all choices of f(x), the unstable fixed points yield comparatively small $L_{\infty }$, even though they are farthest away from the final equilibrium points. It is particularly interesting that $L_{\infty }$ as a function of initial position qualitatively follows f(x), indicating the close connection between the information geometry and the underlying forcing.

Finally, we note that this work can be extended in many different directions, including: (1) If the initial condition is not one Gaussian peak for every period of f(x), but only one peak for some much larger (tending to infinity) number of periods of f(x), then one can study how this initial peak gradually spreads out, how that compares with pure diffusion, and what effect the precise shape of f(x) might have [24,25,26]. This is also related to so-called anomalous diffusion [27,28,29], which can be considerably more general though, involving fractional derivatives. (2) If the force also includes a constant component tending to push the initial condition in a particular direction, it is of interest to study situations where the constant force is comparable to the periodic component of the force [30,31,32]. (3) Allowing the force to vary in time as well as space is relevant to so-called Brownian motors [33,34,35,36]. Work in some of these areas is currently ongoing.

## Appendix A

We now recall how τ(t) and L(t) in Equations (1) and (2) are related to the relative entropy (Kullback–Leibler divergence) [6,11]. We consider two nearby PDFs $p_{1}=p\left(x,t_{1}\right)$ and $p_{2}=p\left(x,t_{2}\right)$ at time $t=t_{1}$ and $t_{2}$, and the limit of a very small $\delta t=t_{2}-t_{1}$ to do Taylor expansion of $D\left[p_{1},p_{2}\right]=\int dx\;p_{2}\ln\left(p_{2}/p_{1}\right)$ by using

(A1) $$\begin{array}{ccc} \frac{\partial }{\partial t_{1}}D\left[p_{1},p_{2}\right] & = & -\int dx\;p_{2}\frac{\partial_{t_{1}}p_{1}}{p_{1}}, \end{array}$$

(A2) $$\begin{array}{ccc} \frac{\partial^{2}}{\partial t_{1}^{2}}D\left[p_{1},p_{2}\right] & = & \int dx\;p_{2}\left\{\frac{{\left(\partial_{t_{1}}p_{1}\right)}^{2}}{p_{1}^{2}}-\frac{\partial_{t_{1}}^{2}p_{1}}{p_{1}}\right\}, \end{array}$$

(A3) $$\begin{array}{ccc} \frac{\partial }{\partial t_{2}}D\left[p_{1},p_{2}\right] & = & \int dx\;\left\{\partial_{t_{2}}p_{2}+\partial_{t_{2}}p_{2}\left[\ln p_{2}-\ln p_{1}\right]\right\}, \end{array}$$

(A4) $$\begin{array}{ccc} \frac{\partial^{2}}{\partial t_{2}^{2}}D\left[p_{1},p_{2}\right] & = & \int dx\;\left\{\partial_{t_{2}}^{2}p_{2}+\frac{{\left(\partial_{t_{2}}p_{2}\right)}^{2}}{p_{2}}+\partial_{t_{2}}^{2}p_{2}\left[\ln p_{2}-\ln p_{1}\right]\right\}. \end{array}$$

In the limit $t_{2}\to t_{1}=t$ ($p_{2}\to p_{1}=p$), Equations (A1)–(A4) give us

(A5) $$\begin{array}{ccc} \lim_{t_{2}\to t_{1}}\frac{\partial }{\partial t_{1}}D\left[p_{1},p_{2}\right] & = & \lim_{t_{2}\to t_{1}}\frac{\partial }{\partial t_{2}}D\left[p_{1},p_{2}\right]=\int dx\partial_{t}p=0,\\ \lim_{t_{2}\to t_{1}}\frac{\partial^{2}}{\partial t_{1}^{2}}D\left[p_{1},p_{2}\right] & = & \lim_{t_{2}\to t_{1}}\frac{\partial^{2}}{\partial t_{2}^{2}}D\left[p_{1},p_{2}\right]=\int dx\frac{{\left(\partial_{t}p\right)}^{2}}{p}=\frac{1}{\tau^{2}}. \end{array}$$

Up to $O({\left(dt\right)}^{2})$ ($dt=t_{2}-t_{1}$), Equation (A5) and $D(p_{1},p_{1})=0$ lead to

(A6) $$D\left[p_{1},p_{2}\right]=\frac{1}{2}\left[\int dx\frac{{\left(\partial_{t}p\left(x,t\right)\right)}^{2}}{p(x,t)}\right]{\left(dt\right)}^{2},$$

and thus the infinitesimal distance $dl(t_{1})$ between $t_{1}$ and $t_{1}+dt$ as

(A7) $$dl\left(t_{1}\right)=\sqrt{D[p_{1},p_{2}]}=\frac{1}{\sqrt{2}}\sqrt{\int dx\frac{{\left(\partial_{t_{1}}p\left(x,t_{1}\right)\right)}^{2}}{p(x,t_{1})}}dt.$$

By summing $dt(t_{i})$ for i=0,1,2,...,n-1 (where n=t/dt) in the limit dt→0, we have

(A8) $$\lim_{dt\to 0}\sum_{i=0}^{n-1}dl\left(idt\right)=\lim_{dt\to 0}\sum_{i=0}^{n-1}\sqrt{D[p(x,idt),p(x,(i+1)]}\;dt\propto \int_{0}^{t}dt_{1}\;\sqrt{\int dx\frac{{\left(\partial_{t_{1}}p\left(x,t_{1}\right)\right)}^{2}}{p(x,t_{1})}}=L\left(t\right),$$

where L(t) is the information length. Thus, L is related to the sum of infinitesimal relative entropy. Note that L is a Lagrangian distance between PDFs at time 0 and *t* and sensitively depends on the particular path that a system passed through reaching the final state. In contrast, the relative entropy D[p(x,0),p(x,t)] depends only on PDFs at time 0 and *t* and thus does not tell us about intermediate states between initial and final states.
